# Exploring the role of ankle torque fluctuations and intramuscular coherence in gait function among older adults

**DOI:** 10.1007/s40520-025-03188-0

**Published:** 2025-09-06

**Authors:** Akira Yaguchi-Horiuchi, Toshiaki Tsuji, Hiroki Takeuchi, Yoshiharu Yokokawa, Eiji Yamanaka, Ippei Nojima

**Affiliations:** 1 Department of Physical Therapy, School of Health Science, Shinshu University, Matsumoto, Japan; 2 Graduate School of Science and Engineering, Saitama University, Saitama, Japan; 3 National Hospital Organization, Higashinagoya National Hospital, Nagoya, Japan; 4 Division of Rehabilitation Sciences, Department of Health Sciences, Medical School, Nagoya City University, Nagoya, Japan

**Keywords:** Ankle torque fluctuations, AR model, Intramuscular coherence, Gait function, Neuromuscular control, Older adults

## Abstract

**Objective:**

This study aimed to investigate the relationship between ankle joint function and walking performance in older adults by assessing qualitative ankle functions through torque fluctuation analysis and tibialis anterior (TA) intramuscular coherence during isometric dorsiflexion.

**Methods:**

Thirty-eight community-dwelling older adults participated in this study. Ankle torque fluctuations and intramuscular coherence were evaluated during a dorsiflexion task at 30% of maximum voluntary torque (MVT). Walking performance was assessed using the 5-meter walk test and the Timed Up and Go (TUG) test. Torque fluctuation indicators, including the coefficient of variation (CV), frequency components, and the primary component calculated by an autoregressive (AR) model, were derived from time-series data. Intramuscular coherence was analyzed in the δ (0–5 Hz) and β (16–35 Hz) frequency bands. Multiple regression analyses adjusted for age were conducted to explore associations between walking performance, torque indicators, and intramuscular coherence.

**Results:**

The TUG test demonstrated a significant relationship with the AR principal component of torque fluctuations, independent of age (*p* = 0.031), suggesting that temporal variability in ankle torque contributes to dynamic balance. While no significant relationship was observed between gait function and intramuscular coherence, δ-band coherence showed significant correlations with torque variability (CV, *r* = 0.598, *p* < 0.001) and spectral power in both the 0.5–5 Hz (*r* = 0.62, *p* < 0.001) and 5–10 Hz (*r* = 0.544, *p* = 0.001) bands.

**Discussion:**

The AR principal component appears to capture kinematic features to motor control and dynamic balance, as evidenced by its association with TUG performance. Furthermore, the relationship between δ-band coherence and torque fluctuations highlights its potential as a maker of neuromuscular function. Although torque fluctuation characteristics and δ-band coherence did not directly correlate with walking speed, they offer valuable insights into the neurophysiological mechanisms underpinning motor control.

**Conclusion:**

This study demonstrated that temporal variability in ankle torque, as quantified by the AR principal component, contributes to walking ability and dynamic balance in older adults.

## Introduction

Gait is a fundamental function for daily living, and its decline significantly affects quality of life [1]. Among older adults, reduced gait ability is linked to higher rates of disease incidence and mortality [2]. Previous studies have demonstrated that older adults with faster walking speeds are more likely to maintain functional independence and experience greater life expectancy [3, 4]. These findings highlight the critical need to identify the physical factors contributing to gait decline in older adults and to implement strategies for preventing gait deterioration, thereby promoting healthy aging and enhancing quality of life in this population.

Among the characteristics of gait in older adults, kinematic changes in joint movements due to aging are particularly pronounced in the ankle joint. Previous studies have reported that ankle dorsiflexion moments and propulsive forces during gait decline with age [5, 6]. Additionally, when walking at the same speed, the proportion of work performed by the muscles around the ankle during the stance phase was 73% in young adults but decreased to 51% in older adults [7]. These findings suggest that the functional role of the ankle joint in walking diminishes with age, underscoring the critical importance of ankle function in the gait of older adults.

Although older adults exhibit these gait characteristics, Franz et al. reported that they can increase their ankle plantarflexor strength by approximately 1.4 times during inclined treadmill walking compared to level walking [8]. This indicates that, despite possessing reserve muscle strength, older adults may not fully utilize it during level walking. Consequently, relying solely on maximal muscle strength assessments may fail to identify older adults with impaired everyday walking performance. Furthermore, while muscle mass—a common metric for skeletal muscle evaluation—was previously regarded as equivalent to muscle strength, recent studies have demonstrated that muscle mass functions as an independent component of motor function [9, 10]. These findings highlight the importance of assessing qualitative aspects of motor function, such as force control and neuromuscular characteristics, rather than relying solely on quantitative metrics like muscle strength and muscle mass, to better explain declines in motor function in older adults.

Reduced stability is a key characteristic of force control in older adults. During isometric contractions at intensities below maximal voluntary contraction (MVC), the exerted force tends to fluctuate [11]. These fluctuations are more pronounced in older adults compared to younger adults [12, 13] and have been found to correlate with walking function [14] and fall history [15]. As a measure of motor control, the coefficient of variation (CV), which normalizes the standard deviation by the mean, is widely used to quantify the magnitude of force fluctuations and is strongly associated with the variance of low-frequency components in synaptic inputs shared among multiple motor neurons [16, 17].

Recently, time-series analysis has been applied to evaluate force control, enabling the quantification of the temporal characteristics of force fluctuations [18–20]. This approach allows to examine the complex dynamics of force production and control over time. While traditional metrics such as CV assume that force fluctuations are random and lack regularity, time-series analysis has demonstrated that force fluctuations possess temporal structures. Analytical methods such as frequency analysis, approximate entropy, and detrended fluctuation analysis have been employed to extract characteristics that cannot be captured solely by the magnitude of force fluctuations [21, 22]. Previous studies have reported a decline in the complexity of force fluctuations in older adults [18], suggesting a diminished ability to appropriately adjust force in response to task demands [23].

Understanding the neurophysiological mechanisms underlying the motor control of force output is essential for comprehending the decline in motor function in older adults. In this context, coherence analysis has recently gained attention as a valuable method for evaluating the activity of the nervous system involved in muscle control. Coherence analysis reflects the functional connectivity between the central and peripheral systems in motor control [24]. Coherence measured between two electromyographic (EMG) signals, either within the same muscle or between different muscles, is thought to represent common synaptic inputs to the motor neuron pool originating from the cerebral cortex and subcortical regions [25–27]. Specifically, beta-band coherence is believed to reflect descending cortical input [28], while delta-band coherence (0–5 Hz) is associated with postural adjustments during standing [29]. Although several studies have reported age-related differences in intramuscular and intermuscular coherence in the beta and delta bands [30–32], the relationships between these coherence measures and motor function or physical characteristics in older adults remain poorly understood.

This study aims to investigate the relationship between ankle force control, considered essential for walking ability in older adults, and walking function by evaluating qualitative ankle functions using force fluctuation metrics from time-series analysis and measures of intramuscular coherence.

## Methods

### Participants

This study included 38 older adults (mean age: 76.2 ± 7.9 years; 16 men and 22 women) aged 60 years or older who participated in specific health checkups organized by local governments and provided informed consent after receiving verbal and written explanations. The study was conducted with approval from the Ethical Review Board of Shinshu University School of Medicine (Approval No. 5548), and written informed consent was obtained from all participants prior to the experiment.

This study was part of a community-based health screening program, with participant recruitment based on the availability of individuals meeting the inclusion criteria during the screening period. A total of 38 eligible older adults were enrolled, reflecting our aim to include as many participants as possible within practical and ethical constraints. Although an a priori power analysis was not feasible due to the opportunistic sampling, the sample size is comparable to or slightly larger than that of similar observational studies in older adults [33, 34] supporting its adequacy for this exploratory investigation.

Participants were excluded if they had a history of orthopedic or neurological disorders affecting the tested lower limb, cognitive impairments, or visual impairments that could hinder their ability to complete the tasks.

### Motor performance

#### Ankle force control task

**Fig. 1:**
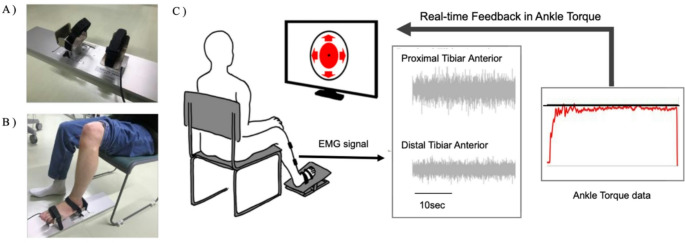
Ankle force control task. **(A)** The measurement device used for assessing ankle dorsiflexion torque, **(B)** The actual setup during measurement, **(C)** Visual feedback being utilized to adjust ankle dorsiflexion torque to match the target value

Participants sat on a chair with their knee and ankle joints flexed at 90°, and their foot securely fixed to a torque sensor. The isometric dorsiflexion torque of the ankle joint, measured using a torque sensor, was displayed in real-time on a PC monitor positioned 1 m away from the participants. Participants performed three trials of maximum voluntary contraction in ankle dorsiflexion, each lasting 3 s, with the Maximum Voluntary Torque (MVT) defined as the highest value recorded across the trials. Subsequently, participants completed dorsiflexion tasks at 30% of their MVT for two 30-second trials, with sufficient rest between trials to prevent fatigue. The target intensity of 30% of MVT was chosen because low-intensity ankle exercises at approximately 30% do not induce severe fatigue while generating adequate muscle activity signals for analysis. Participants were instructed to match and maintain their torque output as accurately and consistently as possible to the 30% MVT target displayed on the PC monitor (Fig. 1). The dominant leg was used for this task, which was identified by asking participants which leg they typically use when kicking a ball.

The dorsiflexion task was chosen as the type of ankle task in this study because it provides more reliable data compared to plantarflexion and due to the significant contribution of the tibialis anterior (TA) muscle to ankle dorsiflexion torque. The TA is responsible for approximately 60% of dorsiflexion torque [35], indicating a strong correlation between dorsiflexion torque fluctuations and the variability in motor neuron firing of the TA [36]. In contrast, ankle plantarflexion torque is generated by approximately eight different muscles, which share a relatively low proportion of common synaptic input [37]. Therefore, the dorsiflexion task is more suitable for examining the relationship between muscle activity and joint torque in contracting muscles.

#### Walking function

Walking function in elderly adults was assessed using the 5-meter walk test and the Timed Up & Go (TUG) test. For the 5-meter walk test, participants were instructed to walk a distance of 5 m at their usual walking pace, and the time taken to complete the distance was recorded. In the TUG test, participants began seated in a standard chair. Upon the examiner’s command, they stood up, walked a distance of 3 m at a comfortable and safe pace, turned around, walked back to the chair, and sat down. The total time required to complete this task was measured in seconds using a stopwatch.

### Data acquisition

#### Ankle torque indicators

Ankle torque data were recorded at a sampling frequency of 30 Hz using a custom-developed torque measurement system [38]. This device was designed to precisely estimate ankle torque by utilizing computational algorithms that mitigate the effects of translational forces and heel movements. For this study, the system was developed using Microsoft Visual Studio (Microsoft), with both the computed torque values and target values displayed on a PC monitor.

#### Muscle activity

Muscle activity was measured using an EMG (A-Cap 4ch, Four Assist) to calculate intramuscular coherence in TA. EMG signals were recorded at a sampling rate of 1000 Hz. Prior to electrode placement, the skin was cleaned with alcohol wipes to reduce impedance to 20 kΩ. Muscle activity from the proximal and distal regions was measured during ankle dorsiflexion force control tasks. The recording sites for the proximal and distal TA electrodes were spaced at least 10 cm apart to minimize the risk of crosstalk between pairs of EMG electrodes.

### Data analysis

The data were analyzed using a custom MATLAB script (MathWorks, Natick, MA, USA), and the following parameters were quantified:

#### Ankle torque indicators

From the 30-second recordings, 20 s of stable torque data were selected from each trial, and the data from two trials were concatenated to obtain 40 s of data for analysis. The difference between the target torque (30% MVT) and the actual torque during the ankle force control task was used as an indicator of motor function. The mean difference from the target were calculated using the following equation:

$$\:Error\:ratio = \:\frac{1}{{\overline x }}\sum {|T - x|} $$

𝑥 represents the exerted torque, $\:\overline x$ is the average torque, and 𝑇 is the target value. The coefficient of variation (CV) of torque was calculated as an indicator of the magnitude of force fluctuations. Additionally, an autoregressive (AR) model was employed to extract features from the time-series characteristics of torque fluctuations. For the AR model, the following AR equation was fitted to the torque data.

$$\begin{gathered}\:{y_t} = \:{\phi _0} + \:{\phi _1}{y_{t - 1}} + \hfill \\{\phi _2}{y_{t - 2}} + \ldots \: + {\phi _p}{y_{t - p}} + \:{\varepsilon _t} \hfill \\ \end{gathered} $$

Here, 𝜑_𝑝_ represents the AR coefficients, 𝑝 is the order, and 𝜀_𝑡_ is the error term. The AR coefficients can be estimated using the Least Squares Method by minimizing the residual errors (*ε*_*t*_). In the analysis of biological signals, selecting the model order solely based on information criteria is not always appropriate. Therefore, the order was set to 10 based on anticipated outcomes for the data used in this study. This choice indicates that the AR model considers data up to 300 ms prior for the current dataset (sampling interval: 30 ms). To reduce dimensionality, principal component analysis (PCA) was applied to the 10 AR coefficients (𝜑₁ to 𝜑₁₀), with the first principal component (PC1) designated as a representative feature of torque fluctuations. For the frequency analysis, a Fast Fourier Transform (FFT) was applied to the torque data. Given that many participants exhibited large amplitude spectra below 10 Hz, this frequency range was divided into two bands: 0.5–5 Hz (Fq0.5-5) and 5–10 Hz (Fq5-10). The total amplitude spectrum for each band was then computed. Subsequently, the Error ratio, CV, PC1, Fq0.5-5, and Fq5-10 derived from the torque data analysis were used as ankle torque indicators. Furthermore, the relationships between the features constituting the principal component derived from PCA and the ankle torque indicators were analyzed.

#### Intramuscular coherence

Intramuscular coherence (|*C*_*s1,s2*_ (*f* )|^2^) was determined using the following equation:

$$\:{\left| {{C_{s1,s2}}\left( f \right)} \right|^2}\: = \:\frac{{{{\left| {{P_{s1,s2}}\left( f \right)} \right|}^2}}}{{|{P_{s1}}\left( f \right)| \cdot |{P_{s2}}\left( f \right)|}}$$

*P*_*s1*_*(f)* and *P*_*s2*_*(f)* represent the auto-spectra of signals *s1* and *s2*, respectively, and *P*_*s1, s2*_*(f)* represents the cross-spectrum at frequency *f*. These spectra were computed by using a discrete Fourier transform to segments of 1024 data points with no overlap. To minimize spectral leakage, a Hanning window with a duration of 1024 ms was applied. The coherence value, which ranges from 0 to 1, was used to quantify the relationship between the two signals: a value of 0 indicates no relationship, while a value of 1 indicates perfectly identical signals. Coherence parameters were computed for two distinct frequency bands: delta (0–5 Hz) and beta (16–35 Hz). The calculated parameters included coherence area. The coherence area was defined as the sum of coherence values exceeding the significance threshold, determined by the 95% confidence interval. The 95% confidence interval was calculated using the formula 1 − (0.05)^[1/(*N*−1)]^, where N represents the number of disjoint segments used in the coherence analysis.

### Statistical analysis

The Shapiro-Wilk test was employed to assess the normality of the data. Correlation analysis was conducted to investigate the relationships between walking function measures (5-meter walk test and TUG) and various parameters, including ankle torque metrics and intramuscular coherence. Pearson’s correlation coefficient was calculated for variables that met the assumption of normality, while Spearman’s rank correlation coefficient was used for variables that did not meet this assumption. To examine the relationships between walking function and ankle torque indicators while accounting for the effect of age, multiple regression analysis was performed using a forced entry method, with walking function (5-meter walk test and TUG) as the dependent variable and ankle torque indicators as independent variables. Since the TUG data did not exhibit normality, a log transformation was applied. To further analyze the factors contributing to each principal component, the correlation between the PC1 loadings and AR coefficients were examined, along with the correlation between the PC1 and the evaluation metrics of ankle joint torque. As a supplementary analysis, additional correlation analyses were conducted to further explore the relationships between ankle torque indicators and intramuscular coherence. These analyses were conducted using R version 4.4.2 and RStudio version 2023.06.0. Statistical significance was set at *p* < 0.05.

## Results

Of the 38 participants, those whose exerted torque deviated by more than ± 50% from the target value during the ankle force control task were excluded from the analysis. As a result, 34 participants were included in the final analysis. Participant characteristics are summarized in Table 1.

**Table 1:**
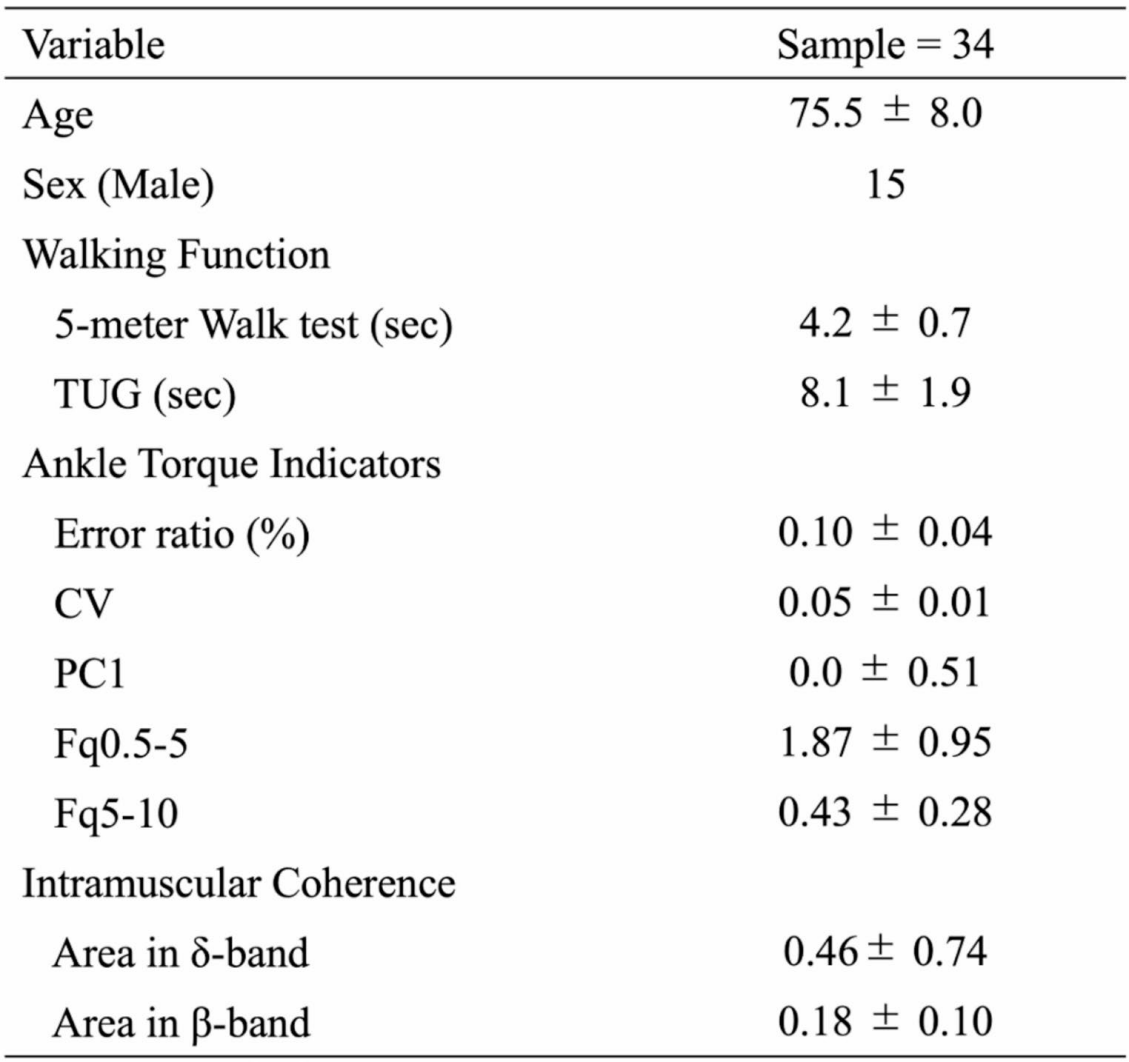
Participant characteristics. This table presents the basic data, task performance, and muscle activity analysis results for the 34 participants included in this study. Statistical significance was set at *p* < 0.05

### Relationship between walking function, ankle torque indicators, and intramuscular coherence

The results of the correlation analysis between walking function, ankle torque indicators, and intramuscular coherence are summarized in Table 2. For walking function, the TUG test demonstrated significant correlations with the Error ratio (*r* = 0.431, *p* = 0.011) and the CV (*r* = 0.397, *p* = 0.02). However, no statistically significant correlations were identified between walking function and the PC1 or intramuscular coherence.

**Table 2:**
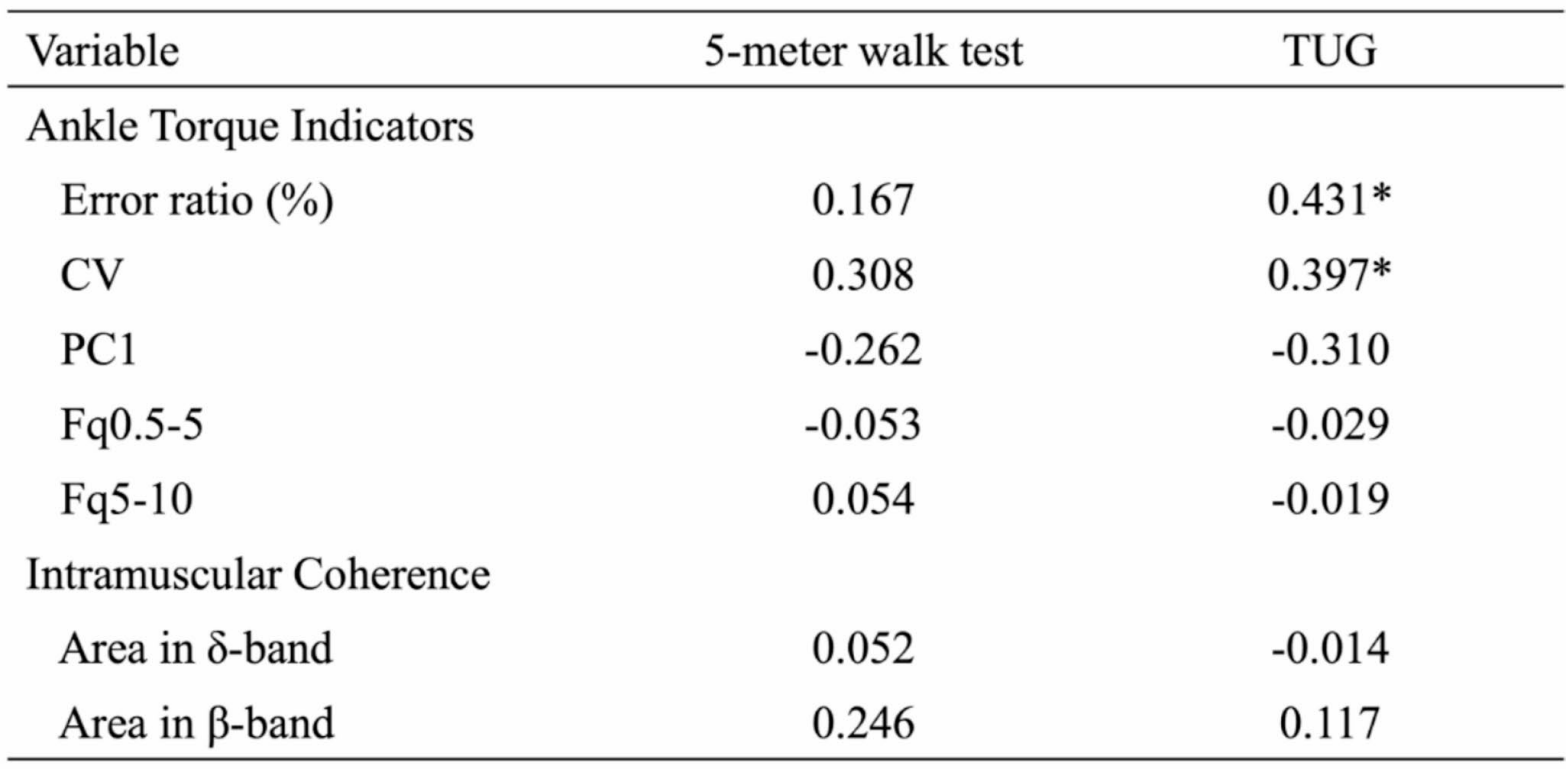
Relationship between walking function, ankle torque indicators, and intramuscular coherence. This table shows the correlations between walking function (5-meter walk test and TUG) and ankle torque indicators as well as intramuscular coherence. The significance level was set at less than 5%, and an asterisk (*) in the table indicates a significant relationship

**Table 3:**
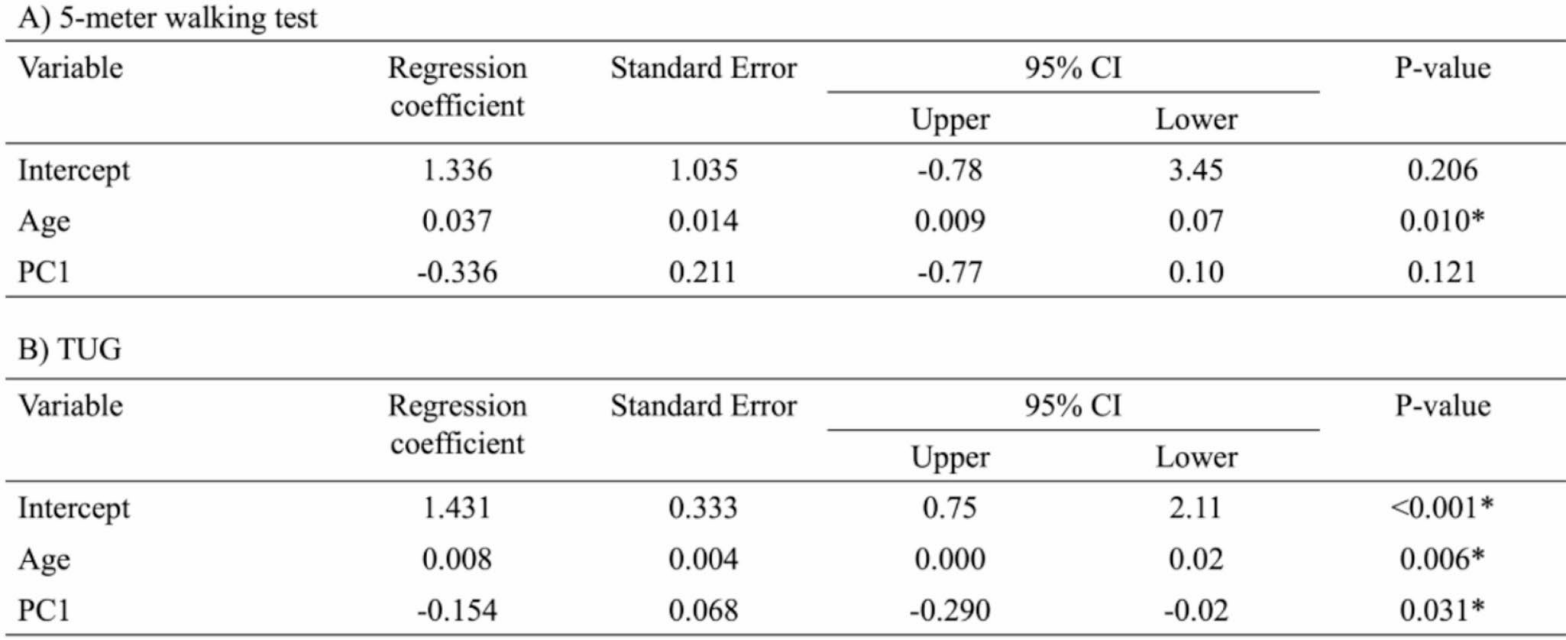
Relationship between walking function and the first principal component. Using age as a covariate, this table displays the relationships between the AR principal component of ankle torque and (A) 5-meter walking test and (B) TUG. The significance level was set at less than 5%, and an asterisk in the table indicates a significant relationship

### Multiple regression analysis of walking function and ankle torque indicators

Table 3A presents the results of the multiple regression analysis with 5-meter walk test as the dependent variable. In this analysis, after adjusting for age as a covariate, the PC1 was not found to be a statistically significant predictor of 5-meter walk test (*p* = 0.121, standardized β = -0.24, 95%CI [-0.55, 0.77], sr^2^ = 0.058, f^2^ = 0.06), indicating a small-to-moderate effect size. Conversely, Table 3B summarizes the multiple regression analysis with TUG as the dependent variable, where the PC1 was identified as a significant predictor of TUG performance (*p* = 0.031, standardized β = -0.34, 95%CI [-0.65, -0.03], sr^2^ = 0.114, f^2^ = 0.13) after accounting for age as a covariate, representing a moderate effect size.

### Factors constituting the first principal component

**Fig. 2:**
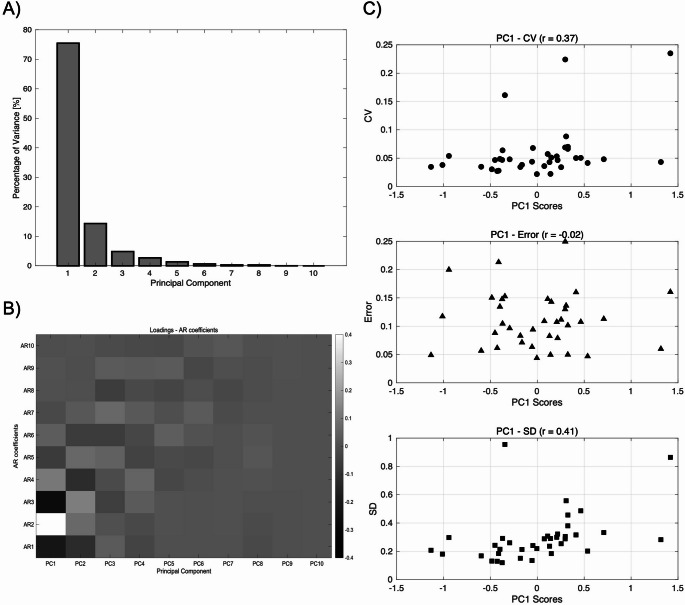
Principal component analysis of AR coefficients. **(A)** The principal component loadings (contributions) are presented, indicating that the first principal component (PC1) explains approximately 75% of the variance, while the first and second principal components combined account for about 90% of the variance. **(B)** The relationship between loadings in each principal component and AR coefficients is depicted, showing that PC1 exhibits strong loadings for lower-order AR coefficients, particularly the 1st to 3rd orders. This suggests that PC1 captures temporal features predominantly associated with low-order autoregressive dynamics in the torque signal. **(C)** The correlation between PC1 and various torque data metrics is illustrated, with significant correlations identified for CV and SD

The PC1 accounted for a high contribution rate of 75% (Fig. 2-A). Analysis of the relationships between the PC1 and AR coefficients revealed that PC1, in particular, exhibited strong correlations with the 1st to 3rd order AR coefficients, suggesting that PC1 is predominantly influenced by lower-order AR coefficients (Fig. 2-B). Moreover, a significant positive correlation was identified between PC1 and the ankle torque metrics, specifically with CV and SD, which are indicators of variability (Fig. 2-C).

### Relationship between ankle torque indicators and intramuscular coherence

The results of the correlation analysis between ankle torque indicators and intramuscular coherence are presented in Table 4. The δ-band coherence area exhibited significant correlations with CV (*r* = 0.598, *p* < 0.001), Fq0.5-5 (*r* = 0.62, *p* < 0.001), and Fq5-10 (*r* = 0.544, *p* = 0.001). Regarding the PC1, no significant correlations were observed with the Error Ratio (*r* = -0.113, *p* = 0.522) or CV (*r* = 0.223, *p* = 0.205), while significant correlations were found with Fq0.5-5 (*r* = 0.697, *p* < 0.001) and Fq5-10 (*r* = 0.642, *p* < 0.001).

**Table 4:**
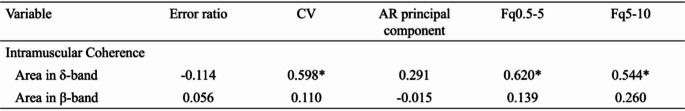
Relationship between ankle torque indicators and intramuscular coherence. This table shows the correlations between intramuscular coherence values calculated from the tibialis anterior and various torque indicators. The significance level was set at less than 5%, and an asterisk in the table indicates a significant relationship

## Discussion

This study aimed to investigate the qualitative function of the ankle joint by analyzing torque fluctuations and intramuscular coherence during an isometric dorsiflexion task in community-dwelling older adults, with the objective of elucidating its association with gait function. The findings demonstrated a significant relationship between TUG performance and the first principal component. Furthermore, δ-band coherence within the TA was significantly correlated with torque variability (CV) and amplitude in specific frequency bands. These results offer valuable insights into the interactions between torque control, neuromuscular function, and walking ability.

### Relationship between walking function and ankle torque indicators

The relationship between gait function and ankle torque indicators was clarified through multivariate analysis adjusted for age. The results revealed a significant negative relationship between TUG and the PC1 (standardized coefficient: -0.154), indicating that the PC1 is significantly associated with walking function. Aging significantly impacts walking function (TUG) [39], potentially obscuring the contribution of the PC1. By including age as a covariate in the analysis, the relationship between TUG and the PC1 was uncovered. This finding suggests that the PC1 may independently influence TUG, regardless of the effects of aging.

Regarding the relationship between the PC1 and other ankle torque indicators, no significant correlations were observed with CV or Error Ratio. the AR model-based assessment of ankle function evaluates factors separate from conventional measures like movement stability and accuracy. Previous study has demonstrated that AR components measured using inertial sensors can classify the presence and severity of ataxic movements with high accuracy [40]. This underscores the potential of AR model-derived features for characterizing individual motor profiles. While the participants in this study exhibited relatively high motor function, and groupings by fall history or frailty were not available, the PC1 still emerged as a promising indicator of ankle function quality. These findings are particularly intriguing as they indicate that the temporal dynamics of ankle motion are not associated with representative values such as CV but are nevertheless closely related to gait performance, similar to those values.

Previous studies have demonstrated a relationship between the CV of TA activity and body sway [41], as well as between the CV of plantarflexor muscle activity and various walking tests [14]. Walking function was evaluated based on the time required to complete walking tests, where faster walking speeds indicate superior walking ability. Propulsive force at the ankle joint has been identified as a critical determinant of walking speed [42] and is closely associated with the activity of muscles such as the gastrocnemius and gluteus maximus [43]. These findings suggest that the features of ankle movements identified by the AR model may represent comprehensive ankle function, even though dorsiflexion torque is not directly responsible for walking speed. Furthermore, while the dynamic contractions of the TA during walking differ in both contraction modality and central control mechanisms from isometric contractions, the results indicate that the CP1 may not only characterize performance during isometric tasks but also reflect the fundamental structural properties of the ankle joint, independent of neuromuscular control strategies. In contrast, no statistically significant relationship was observed between the 5-meter walking time and the PC1. This finding suggests that the PC1 may be more closely associated with dynamic balance than with the walking performance aspects assessed by TUG. Takeuchi et al. [44] reported that muscle strength in TA, rather than plantarflexor strength, plays a critical role in maintaining anterior-posterior stability during cross-stepping movements, highlighting the connection between dynamic balance and TA activity. Given that the TUG serves as a comprehensive assessment of overall motor function, the PC1 may reflect the subtle kinematic features underlying this complex motor ability.

### Interpretation of the first principal component

The first principal component (PC1), derived through PCA during the dimensionality reduction of the 10th-order coefficients calculated using the AR model, was found to be associated with walking function. As shown in Fig. 2(B), the analysis of the relationship between the loadings of each principal component and the AR coefficients revealed that PC1 is correlated with lower-order AR coefficients, suggesting that these coefficients play a significant role in shaping the composition of PC1.

The torque data from the ankle dorsiflexion task exhibit temporal correlations, suggesting that current values can be predicted from past data. Due to its effectiveness in modeling such correlations, the AR model was employed in this study. In its application to biological signals, the AR model has been widely employed for trend evaluation and noise reduction in analyses of electrocardiograms and electroencephalograms [45–47]. Lower-order AR coefficients have been reported to provide an accurate and efficient representation of physiological signals [48]. Furthermore, PC1 exhibited significant correlations with variability indicators such as CV and SD, indicating that it reflects variance that increases over time. This finding suggests that a gradual increase in the variability of ankle joint torque over time may be a contributing factor to the prolongation of TUG time.

### Relationship between δ-Band coherence and ankle torque indicators

No significant relationship was identified between gait function and coherence, which was evaluated to investigate the neurophysiological mechanisms underlying motor function. However, δ-band intramuscular coherence in the TA showed significant correlations with the CV of ankle torque, as well as with Fq0.5-5 and Fq5-10. Previous studies have indicated that δ-band coherence is associated with postural sway [29, 49], which is presumed to result from torque fluctuations. The present results provide directly evidence of a correlation between δ-band coherence and torque fluctuations.

Ankle torque fluctuations have been strongly linked to the variance of low-frequency components of common synaptic inputs to multiple motor neurons [16, 17]. Similarly, δ-band intermuscular coherence has been suggested to reflect shared synaptic inputs among motor neurons [50, 51]. These findings imply that both the 0.5–5 Hz power of ankle torque and δ-band coherence may reflect the common synaptic inputs shared by the motor neuron pool of the TA. Moreover, the spectral power in the 5–10 Hz band (Fq5-10) also showed a positive correlation with δ-band coherence, despite not to overlapping with the frequency range of δ-band coherence. Although it was hypothesized that the frequency characteristics of torque fluctuations would differ between the δ-band and higher frequency ranges, the results suggest that these characteristics are nearly identical. A previous study proposed that the common synaptic inputs to the motor neuron pool contributing to force fluctuations themselves [52]. However, the findings of this study indicate the presence of an alternative mechanism underlying the relationship between intramuscular coherence and motor performance. These results highlight the need for further investigation into the neuromuscular mechanisms driving torque fluctuations.

### Limitations of this study

One limitation of this study is the potential for crosstalk in EMG signals. Although the electrode pairs were positioned as far apart as possible to minimize crosstalk, complete elimination of this issue could not be ensured. Second, accurately assessing common synaptic inputs to motor neurons requires multi-channel EMG analysis. The analysis in this study was restricted to signals obtained from two pairs of electrodes placed on the proximal and distal regions of the TA. Therefore, the presence of common synaptic inputs to motor neurons remains speculative and relies on indirect evaluation metrics. Third, the relationship observed between the PC1of torque and TUG is based on cross-sectional data, leaving the causal nature of this relationship unresolved. To better understand the physical interpretation of the PC1 and its relationship with specific physical functions and characteristics, longitudinal studies are necessary to clarify causality and provide deeper insights.

## Conclusion

This study investigated the qualitative function of the ankle joint in older adults through torque fluctuation analysis and TA intramuscular coherence during isometric dorsiflexion. A significant age-independent negative relationship was identified between TUG and the PC1, suggesting that reduced time-series variability in ankle function contributes to walking ability and dynamic balance. Furthermore, δ-band coherence showed a positive correlation with torque fluctuation metrics, indicating that greater shared synaptic input to the motor neuron pool may contribute to increased variability in torque output. These findings underscore the critical role of neuromuscular control in walking function and offer valuable insights for developing targeted rehabilitation strategies to improve mobility and reduce fall risk in older adults.

## Data Availability

The datasets generated and/or analyzed during the current study are not publicly available due to ethical considerations, but are available from the corresponding author on reasonable request.
